# Supplementary material for: Empirical methods for controlling false positives and estimating confidence in ChIP-Seq peaks
Source: BMC Bioinformatics. 2008 Dec 5;9:523. doi: 10.1186/1471-2105-9-523 (PMC2628906; doi:10.1186/1471-2105-9-523)
Supplement: Additional File 1 — A variety of html documents from the USeq web site detailing the available applications, their best usage, output file type descriptions, command line menus, etc. [file 1471-2105-9-523-S1.zip › USeqUserGuides/JavaDoc.zip!/JavaDoc/index-files/index-5.html]

E-Index


|  |  |  |
| --- | --- | --- |
| **Overview** \| Package \| Class \| Use \| **Tree** \| **Deprecated** \| **Index** \| **Help** | |  |
| **PREV LETTER**   **NEXT LETTER** | **FRAMES**    **NO FRAMES**     **All Classes** |  |


A B C D E F G H I J L M N O P Q R S T U V W X Z 

---


## **E**

**edu.utah.seq.analysis** - package edu.utah.seq.analysis: Primary analysis applications and helper classes for working with binary PointData, xxx.bar.zip files. **edu.utah.seq.compression** - package edu.utah.seq.compression: **edu.utah.seq.data** - package edu.utah.seq.data: Helper classes for working with binary PointData, EnrichedRegions, etc. **edu.utah.seq.methylation454** - package edu.utah.seq.methylation454: Applications for working with 454 data and identifying bisulfite converted bases for methylation studies. **edu.utah.seq.misc** - package edu.utah.seq.misc: **edu.utah.seq.parsers** - package edu.utah.seq.parsers: Parsers for converting sequencing data into binary PointData, xxx.bar.zip files. **ElandExportLine** - Class in edu.utah.seq.parsers: **ElandExportLine(String, int, float)** - Constructor for class edu.utah.seq.parsers.ElandExportLine: Parses an Eland Extended output line, will shift the stranded position by the offSet. **ElandExportLine(String, int[][])** - Constructor for class edu.utah.seq.parsers.ElandExportLine: Stripped constructor/ method for ElandSequenceParser **ElandExportLine(String, int, boolean)** - Constructor for class edu.utah.seq.parsers.ElandExportLine: For parsing a stand alone Eland run. **ElandMultiParser** - Class in edu.utah.seq.parsers: Parses an Eland xxx.eland\_multi.txt file tabulating hits to each fasta entry. **ElandMultiParser(String[])** - Constructor for class edu.utah.seq.parsers.ElandMultiParser: **ElandParser** - Class in edu.utah.seq.parsers: Parses an Eland extended \_export.txt or \_sorted.txt file into point data, split by chromosome and strand. **ElandParser(String[])** - Constructor for class edu.utah.seq.parsers.ElandParser: **ElandSequenceParser** - Class in edu.utah.seq.parsers: Parses an Eland Extended xxx.export.txt or xxx.sorted.txt for sequencing information. **ElandSequenceParser(String[])** - Constructor for class edu.utah.seq.parsers.ElandSequenceParser: **Email** - Class in util.gen: **Email(String, String, String, String)** - Constructor for class util.gen.Email: **engineDigest()** - Method in class util.gen.MD5: **engineGetDigestLength()** - Method in class util.gen.MD5: **engineReset()** - Method in class util.gen.MD5: **engineUpdate(byte)** - Method in class util.gen.MD5: **engineUpdate(byte[], int, int)** - Method in class util.gen.MD5: **EnrichedRegion** - Class in edu.utah.seq.data: **EnrichedRegion(SmoothingWindow, Info)** - Constructor for class edu.utah.seq.data.EnrichedRegion: **EnrichedRegion(SmoothingWindow, int)** - Constructor for class edu.utah.seq.data.EnrichedRegion: **EnrichedRegion()** - Constructor for class edu.utah.seq.data.EnrichedRegion: **EnrichedRegionMaker** - Class in edu.utah.seq.analysis: Collapses an array of window into and array of enriched regions based on score and max gap. **EnrichedRegionMaker(int, int, SmoothingWindowInfo[])** - Constructor for class edu.utah.seq.analysis.EnrichedRegionMaker: **EnrichedRegionMaker(String[])** - Constructor for class edu.utah.seq.analysis.EnrichedRegionMaker: **Enzyme** - Class in util.bio.digest: Restriction enzyme information. **Enzyme(String, String, String, String, String)** - Constructor for class util.bio.digest.Enzyme: **ENZYMES** - Static variable in class bioroot.oligo.OligoBase: **estimateExtensions()** - Method in class trans.misc.CalculateGeneExtensions: **estimatePValue(double)** - Static method in class util.bio.calc.Alignment: Calculates the standard normal cumulative distribution given a z-ratio. **executeCommandLine(String[])** - Static method in class util.gen.IO: Executes tokenized params on command line, use full paths. **executeCommandLine(String[], File)** - Static method in class util.gen.IO: Executes tokenized params on command line, use full paths. **executeCommandLine(String[], File, String[])** - Static method in class util.gen.IO: Executes tokenized params on command line, use full paths. **executeCommandLineReturnAll(String[])** - Static method in class util.gen.IO: Executes tokenized params on command line, use full paths. **executeRScript()** - Method in class util.bio.wrappers.Segment: **executeShellScript(String, File)** - Static method in class util.gen.IO: Executes a String of shell script commands via a temp file. **executeSQL(String)** - Method in class bioroot.DBUtil: **executeSQL(String)** - Method in class util.gen.SQL: **executeSQLAdvance(String)** - Method in class bioroot.DBUtil: Execute and advance the ResultSet. **executeSQLAdvance(String)** - Method in class util.gen.SQL: Execute and advance the ResultSet. **executeSQLUpdate(String)** - Method in class bioroot.DBUtil: Fires SQL Update statement returning true if no problems were encountered. **executeSQLUpdate(String)** - Method in class util.gen.SQL: Fires SQL Update statement returning true if no problems were encountered. **ExonIntron** - Class in util.bio.annotation: ExonIntron info. **ExonIntron(int, int)** - Constructor for class util.bio.annotation.ExonIntron: **ExonIntron(int, int, String)** - Constructor for class util.bio.annotation.ExonIntron: **ExonIntron(Gff3Feature)** - Constructor for class util.bio.annotation.ExonIntron: **ExonScoreExtractor** - Class in trans.roc: Extracts scores from xxx.bar files for particular groups of exons. **ExonScoreExtractor(String[])** - Constructor for class trans.roc.ExonScoreExtractor: **expandFlatTopPeak()** - Method in class trans.main.PeakPicker: Attempts to expand the leftIndex and rightIndex **expandFloatArray(float[], int, int)** - Static method in class util.gen.Num: Converts a float[] to float[][], must be certain about the number of rows and colmns! **ExportExons** - Class in util.bio.annotation: **ExportExons(File)** - Constructor for class util.bio.annotation.ExportExons: **ExportIntergenicRegions** - Class in util.bio.annotation: **ExportIntergenicRegions(String[])** - Constructor for class util.bio.annotation.ExportIntergenicRegions: **exportIntergenicRegions()** - Method in class util.bio.annotation.ExportIntergenicRegions: **ExportIntervalData** - Class in trans.main: **ExportIntervalData(String[])** - Constructor for class trans.main.ExportIntervalData: **ExportIntronicRegions** - Class in util.bio.annotation: **ExportIntronicRegions(String[])** - Constructor for class util.bio.annotation.ExportIntronicRegions: **expr** - package expr: Classes and tools related to expression array analysis. **ExpressedGene** - Class in expr: **ExpressedGene(String, Coordinate, float[])** - Constructor for class expr.ExpressedGene: **ExpressionScores2CoordinatesViaGFF** - Class in expr: **ExpressionScores2CoordinatesViaGFF()** - Constructor for class expr.ExpressionScores2CoordinatesViaGFF: **extract(File, boolean)** - Method in class util.bio.parsers.gff.DmelRel4Extractor: **extract1lqFile(File, File)** - Method in class trans.qc.CoordinateExtractor1lq: Fetches coordinates for DESTYPEs of 0, 3, -3, 6, or -6 (Control) and PM -111, 111. **extract1lqFile(File, File)** - Static method in class trans.qc.Parser1lq: Fetches coordinates for DESTYPEs of 0, 3, -3, 6, or -6 (Control) and PM -111, 111. **extract1lqFiles()** - Method in class trans.qc.CoordinateExtractor1lq: Extracts control oligo information from 1lq files. **extract1lqFiles()** - Method in class trans.qc.Parser1lq: Extracts control oligo information from 1lq files. **extractCGNames(ArrayList)** - Static method in class trans.anno.AnnotateRegions: Extracts the names of each gene group returning an ArrayList of Strings. **extractCGNames(ArrayList)** - Static method in class trans.anno.AnnotateRegionsWithGeneList: Extracts the names of each gene group returning an ArrayList of Strings. **extractChrFiles(File)** - Static method in class trans.tpmap.WindowMaker: **extractChromosomeName(String)** - Static method in class util.bio.seq.Seq: Attempts to extract chr1,2,3...22 or chrX,Y,M,MT from the String. **extractFileName(String)** - Static method in class meme.UtilMeme: **extractFiles(File)** - Static method in class util.gen.IO: Extracts the full path file names of all the files and directories in a given directory. **extractFiles(File, String)** - Static method in class util.gen.IO: Extracts the full path file names of all the files in a given directory with a given extension (ie txt or .txt). **extractFiles(String)** - Static method in class util.gen.IO: Extracts all files from a comma delimited string of files and directories. **extractFiles(String, String)** - Static method in class util.gen.IO: Extracts all files with a given extension from a comma delimited string of files and directories. **extractFiles(String[], String)** - Static method in class util.gen.IO: Given full path file or directory names, extracts all files given an extension. **extractFilesReturnFiles(File, String)** - Static method in class util.gen.IO: Extracts the full path file names of all the files in a given directory with a given extension. **extractFilesStringNames(File, String)** - Static method in class util.gen.IO: Extracts the full path file names of all the files in a given directory with a given extension. **extractGeneGroups(HashMap, String[])** - Static method in class trans.roc.ParsePatternedWindows: Pulls out a sub set of GeneGroups with the names in the geneList. **extractGeneGrps(ArrayList, boolean)** - Static method in class trans.anno.BindingRegion: Returns summary line or name for each GeneGrp in ArrayList **ExtractGffFeatures** - Class in util.bio.parsers.gff: **ExtractGffFeatures(String[])** - Constructor for class util.bio.parsers.gff.ExtractGffFeatures: **extractGffFeatures()** - Method in class util.bio.parsers.gff.ExtractGffFeatures: **extractIdFromConcat(String)** - Static method in class util.gen.Misc: Takes a String like testSlide:33328:testslide desc...:1 or test: gip: 2, splits on the : and looks for ID in the last String[x], if not found it returns zero, otherwise it returns a parsed int. **extractIdFromFrontOfConcat(String)** - Static method in class util.gen.Misc: Takes a String like 1: testSlide: 33328:testslide desc etc or 2: test: gip, splits on the : and looks for ID in the first String[x], if not found it returns zero, otherwise it returns a parsed int. **extractIDsFromConcats(String[])** - Static method in class util.gen.Misc: Ditto to extractIDFromConcat but works on String[] returning IDs as a String[]. **extractLogin(String)** - Static method in class bioroot.AccountManagement: **ExtractNonOverlappingWindows** - Class in trans.roc: Parses out non overlapping `RocWindow`s. **ExtractNonOverlappingWindows(File)** - Constructor for class trans.roc.ExtractNonOverlappingWindows: **extractNonOverlappingWindows(RocWindow[])** - Static method in class trans.roc.ExtractNonOverlappingWindows: Assumes the windows are sorted **extractNormalizedValues()** - Method in class trans.cel.QuantileNormalization: Extracts out the normalized values regenerating the original float[][] except the scores have been quantile normalized. **extractOnlyDirectories(File)** - Static method in class util.gen.IO: Returns directories or null if none found. **extractOnlyFiles(File)** - Static method in class util.gen.IO: Extracts the full path file names of all the files not directories in a given directory. **extractPositionScores(Point[])** - Static method in class edu.utah.seq.data.Point: **ExtractSaveSgrScores** - Class in trans.roc: Application for extracting and saving the scores from a .sgr file as a serialized float[]. **ExtractSaveSgrScores(String[])** - Constructor for class trans.roc.ExtractSaveSgrScores: **extractScores()** - Method in class trans.roc.ExonScoreExtractor: **ExtractScoresForRoc** - Class in trans.roc: Assignes values from an sgr file to a collection of `RocWindow`s. **ExtractScoresForRoc(String[])** - Constructor for class trans.roc.ExtractScoresForRoc: **extractSegs(ArrayList)** - Method in class selex.SeqRead: **extractStartEnds(ExonIntron[])** - Static method in class gata.main.GATAUtil: Deep copies start stops from ExonIntron objects to an ArrayList **extractStartEnds(ExonIntron[])** - Static method in class util.bio.annotation.GeneGroup: **extractStartEndsInts(ExonIntron[])** - Static method in class gata.main.GATAUtil: **extractStartEndsInts(ExonIntron[])** - Static method in class util.bio.annotation.GeneGroup: **extractSubRegionOligos(int, int)** - Method in class trans.main.Interval: Given a bp start and bp end as well as a larger Oligo[] returns a sub array of the oligos flanking and including the start and end. **extractType()** - Method in class bioroot.UserRegistrationBean: **ExtractWords** - Class in bioroot.parsers: **ExtractWords(String[])** - Constructor for class bioroot.parsers.ExtractWords

---


|  |  |  |
| --- | --- | --- |
| **Overview** \| Package \| Class \| Use \| **Tree** \| **Deprecated** \| **Index** \| **Help** | |  |
| **PREV LETTER**   **NEXT LETTER** | **FRAMES**    **NO FRAMES**     **All Classes** |  |


A B C D E F G H I J L M N O P Q R S T U V W X Z 

---
